# Lion’s Mane Mushroom, *Hericium erinaceus* (Bull.: Fr.) Pers. Suppresses H_2_O_2_-Induced Oxidative Damage and LPS-Induced Inflammation in HT22 Hippocampal Neurons and BV2 Microglia

**DOI:** 10.3390/antiox8080261

**Published:** 2019-08-01

**Authors:** Naufal Kushairi, Chia Wei Phan, Vikineswary Sabaratnam, Pamela David, Murali Naidu

**Affiliations:** 1 Mushroom Research Centre, Institute of Biological Sciences, Faculty of Science, University of Malaya, Kuala Lumpur 50603, Malaysia; 2 Department of Anatomy, Faculty of Medicine, University of Malaya, Kuala Lumpur 50603, Malaysia; 3 Department of Pharmacy, Faculty of Medicine, University of Malaya, Kuala Lumpur 50603, Malaysia

**Keywords:** *Hericium erinaceus*, mushroom, neuroprotection, anti-inflammation, antioxidants

## Abstract

Oxidative stress and inflammation in neuron-glia system are key factors in the pathogenesis of neurodegenerative diseases. As synthetic drugs may cause side effects, natural products have gained recognition for the prevention or management of diseases. In this study, hot water (HE-HWA) and ethanolic (HE-ETH) extracts of the basidiocarps of *Hericium erinaceus* mushroom were investigated for their neuroprotective and anti-inflammatory activities against hydrogen peroxide (H_2_O_2_)-induced neurotoxicity in HT22 mouse hippocampal neurons and lipopolysaccharide (LPS)-induced BV2 microglial activation respectively. HE-ETH showed potent neuroprotective activity by significantly (*p* < 0.0001) increasing the viability of H_2_O_2_-treated neurons. This was accompanied by significant reduction in reactive oxygen species (ROS) (*p* < 0.05) and improvement of the antioxidant enzyme catalase (CAT) (*p* < 0.05) and glutathione (GSH) content (*p* < 0.01). Besides, HE-ETH significantly improved mitochondrial membrane potential (MMP) (*p* < 0.05) and ATP production (*p* < 0.0001) while reducing mitochondrial toxicity (*p* < 0.001), Bcl-2-associated X (Bax) gene expression (*p* < 0.05) and nuclear apoptosis (*p* < 0.0001). However, gene expression of Nuclear factor erythroid 2-related factor 2 (Nrf2), heme oxygenase 1 (HO-1) and NAD(P)H quinone dehydrogenase 1 (NQO1) were unaffected (*p* > 0.05). HE-ETH also significantly (*p* < 0.0001) reduced nitric oxide (NO) level in LPS-treated BV2 indicating an anti-inflammatory activity in the microglia. These findings demonstrated HE-ETH maybe a potential neuroprotective and anti-inflammatory agent in neuron-glia environment.

## 1. Introduction

Oxidative stress is closely related to the pathogenesis of neurodegenerative disorders, including Alzheimer’s and Parkinson’s diseases [1,2]. The underlying molecular mechanisms include accumulation of reactive oxygen species (ROS) and reactive nitrogen species (RNS) which consequently leads to lipid, protein, and organelle damage, mitochondrial membrane collapse and apoptosis that culminates in neuronal death [3,4]. There is growing evidence that inflammatory reactions and the release of pro-inflammatory cytokines also result in oxidative stress and redox homeostasis disruption associated to mitochondrial dysfunction [5,6]. Excessive generation of ROS and RNS (H_2_O_2_, O_2_^−^, NO, ONOO^−^/ONOOH) can easily lead to the neuronal cell injury because these cells possess low oxidative resistance, high metabolic capacity, and non-replicative nature [7].

Synthetic neuroprotective agents used to treat neurodegenerative diseases have side effects such as dry mouth, tiredness, drowsiness, anxiety, and difficulty with balance [8]. Therefore, natural product-based nutraceuticals or functional food with potent antioxidant and anti-inflammatory activities are of special interest for the development of neuroprotective therapeutics that are both effective and well tolerated. Numerous studies have well-documented mushrooms, including *Ganoderma lucidum* (M.A. Curtis: Fr.) P. Karst [9], *Cyathus striatus* (Huds.) Willd. [10], *Flammulina velutipes* (Curtis) Singer [11], and *Pleurotus ostreatus* (Jacq.) P. Kumm. [12] as potential candidates in combatting neurodegenerative diseases. *Hericium erinaceus* (Bull.:Fr.) Pers., or its common names, Lion’s mane or Monkey’s head mushroom, is a well-established culinary and medicinal mushroom for brain and nerve health. Hericenones (meroterpenoids) and erinacines (cyathane diterpenoids) are the two important classes of compounds isolated from *H. erinaceus* proven to induce the biosynthesis of nerve growth factor (NGF) in nerve cells in vitro [13,14] Previous studies have shown that *H. erinaceus* possessed potent antioxidant activities [15,16,17,18]. In vitro neuroprotection of *H. erinaceus* was demonstrated in several studies against amyloid beta [19,20], 1-methyl-4-phenyl-1,2,3,6-tetrahydropyridine (MPTP) [21] and glutamate-induced neurotoxicity [22,23]. Meanwhile, anti-inflammatory activities of *H. erinaceus* were vastly reported in RAW 264.7 murine macrophage from blood [24,25,26,27,28].

There is currently no report on the neuroprotective effects of *H. erinaceus* in H_2_O_2_-induced neurotoxicity. One study carried out in NG108-15 neuroblastoma–glioma cell line using hot water extract of *H. erinaceus,* was found to be non-beneficial [29]. Similarly, anti-inflammatory effects of *H. erinaceus* in brain microglia has never been reported. As numerous studies demonstrated the activities in RAW 264.7 murine macrophages, *H. erinaceus* may have anti-neuro-inflammatory activities. As more studies reported neuro-health promotion by the extracts or compounds, especially erinacines isolated from the mycelia of the mushroom, it is worthy to investigate the fruiting bodies (basidiocarps), the mushroom part where hericenones are found [30]. Hence, the current study focused on the extracts of the basidiocarps of *H. erinaceus* for neuroprotective activities in H_2_O_2_-induced neurotoxicity of HT22 hippocampal neurons. Both hot water (HE-HWA) and ethanolic (HE-ETH) extracts were also studied for their anti-inflammatory activities in LPS-induced inflammation in BV2 microglia to mimic the inflammation in the brain. Further, the mechanisms of neuroprotection were elucidated via investigation of antioxidant, anti-apoptosis, and mitochondrial functioning.

## 2. Materials and Methods

### 2.1. Chemicals and Reagents

Dulbecco’s modified Eagle’s Medium (DMEM), fetal bovine serum (FBS), penicillin/streptomycin (P/S) and TrypLE™ Express were obtained from GIBCO/Life Technologies (Grand Island, NY, USA). Hydrogen peroxide (H_2_O_2_), lipopolysaccharides (LPS), Griess reagent, sodium nitrite, gallic acid, Folin–Ciocalteu’s phenol reagent (FC reagent), sodium carbonate anhydrous (Na_2_CO_3_) 2,2-diphenyl-1-picrylhydrazyl (DPPH) and 2’,7’-Dichlorodihydrofluorescin diacetate (DCFH-DA) were purchased from Sigma-Aldrich (St. Louis, MO, USA). Tetramethylrhodamine ethyl ester (TMRE) and Hoechst 33258 were purchased from Enzo Life Sciences and Biotium (Hayward, CA, USA) respectively.

### 2.2. Preparation of Hot Water and Ethanolic Extracts

Fresh basidiocarps of *Hericium erinaceus* obtained from Ganofarm Sdn. Bhd., Tanjung Sepat, Malaysia were freeze-dried, blended, and stored at −20 °C prior to the extraction. The hot water extract was prepared as reported previously [31].The freeze-dried powder was dissolved in distilled water at a ratio of 1 : 20 (*w*/*v*) and left for 24 h at 27 ± 2 °C at 150 rpm. Then the mixture was double boiled for 30 min, cooled, and filtered. The aqueous extract was then freeze-dried at −50 ± 2 °C for 48 h.

The ethanolic extract was prepared as described previously [30]. The freeze-dried powder was soaked in 80% (*v*/*v*) aqueous ethanol at a ratio of 1: 10 (*w/v*) for three days at room temperature. At one-day interval, the solvent containing extract was decanted, filtered and the residue was re-soaked in 80% (*v*/*v*) aqueous ethanol. The extraction and filtration process were repeated for another two times. The solvent containing extract was then pooled and concentrated under vacuum using a rotary evaporator to give the ethanolic extract. Both hot water (HE-HWA) and ethanolic (HE-ETH) extracts were stored at −20 °C prior to assay.

### 2.3. Estimation of Total Phenolic Content (TPC)

TPC was measured as reported previously [32]. Briefly, 50 μL of HE-HWA and HE-ETH or gallic acid standard solution was mixed with 50 μL of 10% FC reagent. Then, 100 μL of 10% (*w*/*v*) sodium carbonate solution was added. The mixture was then incubated for one hour in the dark and the absorbance was measured at 750 nm using microplate reader (Sunrise, Tecan, Austria). Results were expressed as mg gallic acid equivalents (GAE) per gram of sample.

### 2.4. 2,2-Diphenyl-1-Picrylhydrazyl (DPPH) Free Radical Scavenging Assay

DPPH free radical scavenging activity was measured as described previously [33]. DPPH reagent prepared in absolute ethanol (100 μM; 90 μL) was added to 10 μL of HE-HWA and HE-ETH at final concentration of 1 mg/mL. The mixture was incubated for 30 min in the dark and the absorbance was then measured at 518 nm. Distilled water was used as the control. The percentage inhibition or scavenging effect was calculated using the formula below:

DPPH radical scavenging (%) = ((Absorbance of control − Absorbance of sample)/Absorbance of control) × 100

### 2.5. Cell Culture

HT22 mouse hippocampal neurons were obtained from Thermo Fisher Scientific Inc. (Waltham, MA, USA) and BV2 murine microglia were a kind gift from Sharmili Vidyadaran, Universiti Putra Malaysia, Selangor, Malaysia. The cells were maintained in DMEM with 10% FBS and 1% P/S at 37 °C, 5% CO_2_, and 90% humidity. At confluency, they were detached by TrypLE™ Express for assays and sub-culturing.

### 2.6. MTS Cell Viability Assay

HT22 cells were plated at a density of 1 × 10^4^ cells per well in 96-well plates and incubated overnight at 37 °C in a 5% CO_2_-humidified incubator. Then, the medium was replaced with HE-HWA or HE-ETH (0 μg/mL, 50 μg/mL, 100 μg/mL, 200 μg/mL, and 400 μg/mL) with or without 250 μM of H_2_O_2_ in complete DMEM medium. BV2 cells were plated at a density of 5 × 10^4^ cells per well in 96-well plates and incubated overnight at 37 °C in a 5% CO_2_-humidified incubator. Then, the medium was replaced with HE-HWA or HE-ETH (0 μg/mL, 50 μg/mL, 100 μg/mL, 200 μg/mL, and 400 μg/mL) with or without 1 μg/mL of LPS in complete DMEM medium. After 24 h of incubation, cell viability was measured using CellTiter 96 s AQ_ueous_ Non-Radioactive Cell Proliferation Assay kit (Promega, Madison, WI, USA). In brief, after the indicated treatment, 20 μL MTS (3-(4,5-dimethylthiazol-2-yl)-5-(3-carboxymethoxyphenyl)-2-(4-sulfophenyl)-2H-tetrazolium) solution was added to each well. Plates were incubated for an additional 4 h at 37 °C, after which the optical density was measured at 490 nm.

### 2.7. Nitric Oxide (NO) Level Assay

BV2 cells were seeded on a 96 well plate (5 × 10^4^ cells/well) and treated with 1 μg/mL LPS in the presence or absence of HE-HWA or HE-ETH for 24 h. The concentration of nitrite (NO_2_), a soluble oxidation product of NO, in the culture media was measured using Griess reagent (0.1% N-1-napthylethylenediamine dihydrochloride and 1% sulfanilamide in 5% phosphoric acid). Fifty microliters of supernatant were mixed with an equal volume of the Griess reagent and optical density was measured at 540 nm. The resulting NO level was interpreted by comparing the absorbance with the sodium nitrite standard curve.

### 2.8. Reactive Oxygen Species (ROS) Assay

HT22 cells were plated at a density of 1 × 10^4^ cells per well in 96-well plate and incubated overnight at 37 °C in a 5% CO_2_-humidified incubator before treatment. After 6 h of treatment, media was removed and the cells were loaded with 10 μM DCFH-DA in phosphate buffered saline (PBS). The plate was returned to 37 °C incubator and incubated for 20 min. After washing with PBS, images were obtained using fluorescence microscope (Nikon Ti-S eclipse, Melville, NY, USA) and fluorescence intensity was measured at an excitation wavelength of 495 nm and an emission wavelength of 529 nm in a multi-mode reader (Biotek^TM^ Synergy H1 Hybrid Multi-Mode Reader, Winooski, VT, USA).

### 2.9. Tetramethylrhodamine Ethyl Ester (TMRE) Staining of Mitochondrial Membrane Potential (MMP)

HT22 cells were plated at a density 1 × 10^4^ cells per well in 96-well plate and incubated overnight at 37 °C in a 5% CO_2_-humidified incubator before treatment. After 6 h of treatment, media was removed, and the cells were loaded with 1 μg/mL of TMRE in PBS. The plate was returned to 37 °C incubator and incubated for 20 min. After washing with PBS, images were obtained using fluorescence microscope and fluorescence intensity was measured at an excitation wavelength of 549 nm and an emission wavelength of 575 nm.

### 2.10. Hoechst 33258 Nuclear Apoptotic Staining

HT22 cells were plated at a density of 1 × 10^4^ cells per well in 96-well plate and incubated overnight at 37 °C in a 5% CO_2_-humidified incubator before treatment. After 6 h of treatment, media was removed, and the cells were loaded with 10 μg/mL of Hoechst 33,258 in PBS. The plate was returned to 37 °C incubator and incubated for 20 min. After washing with PBS, images were obtained with fluorescence microscope. Neurons with fragmented or condensed DNA and neurons with normal DNA were counted in three different fields (containing about 200–250 cells each) per well and the data was presented as apoptotic neurons as a percentage of total neurons.

### 2.11. Measurement of Catalase (CAT) and Glutathione (GSH) Level

The activity of antioxidant enzyme CAT and GSH content in HT22 cells were determined using commercially available kits obtained from Cayman Chemical (Ann Arbor, MI, USA). The kit was used in accordance with the manufacturer’s instructions and the resulting absorbances were measured using microplate reader.

### 2.12. Measurement of Mitochondrial Toxicity and Adenosine Triphosphate (ATP) Levels

The levels of mitochondrial toxicity and ATP indicating mitochondrial functioning in HT22 cells were measured using Mitochondrial ToxGlo™ Assay kit from Promega™. The kits were used in accordance with the manufacturer’s instructions. The resulting fluorescence and luminescence were measured in a multi-mode reader.

### 2.13. Measurement of Caspase 3 Activity

The activity of caspase 3 indicating apoptosis in HT22 cells was measured using Caspase-3 Assay Kit from Abcam (Cambridge, UK). The kits were used in accordance with the manufacturer’s instructions. The resulting absorbances were measured using microplate reader.

### 2.14. Quantitative Polymerase Chain Gene (qPCR) Expression Analysis

Total RNA was obtained from the cultured HT22 neurons using FavorPrep^TM^ Total RNA purification mini kit according to the manufacturer’s protocol (Favorgen Biotech Corp., Pingtung, Taiwan). Reverse transcription and qPCR were performed using the SensiFAST Hi-ROX One-Step mastermix (BioLine, London, UK) in StepOnePlus Real-Time PCR instrument (Applied Biosystems, Foster City, CA, USA). The gene expression levels of Nuclear factor erythroid 2-related factor 2 (Nrf2), Heme oxygenase-1 (HO-1), NAD(P)H quinone dehydrogenase 1 (NQO1), B-cell lymphoma 2 (Bcl-2) and Bcl-2-associated X (Bax) were calculated according to the relative quantitative 2^−ΔΔCt^ method normalized with Glyceraldehyde 3-phosphate dehydrogenase (GAPDH) as the housekeeping gene. The PCR primer sequences used were as follows:
Nrf2:  Forward 5′-TCTCCTCGCTGGAAAAAGAA-3′  Reverse 5′-AATGTGCTGGCTGTGCTTTA-3′HO-1:  Forward 5′-AGG TGT CCA GAG AAG GCT T-3′  Reverse 5′-ATC TTG CAC CAG GCT AGC A-3′NQO1:  Forward 5′-ATC CTT CCG AGT CAT CTC TA-3′  Reverse 5′-CAA CGA ATC TTG AAT GGA GG-3′Bcl-2:  Forward 5′-TCGCAGAGATGTCCAGTCAG-3′  Reverse 5′-ATGCCGGTTCAGGTACTCAG-3′Bax:  Forward 5′-CACAGCGTGGTGGTACCTTA-3′  Reverse 5′-TCTTCTGTACGGCGGTCTCT-3′GAPDH:  Forward 5′-TCACCACCATGGAGAAGGC-3′  Reverse 5′-GCTAAGCAGTTGGTGGTGCA-3′


### 2.15. Statistical Analysis

Data were presented as mean of triplicates ± SD. Three independent experiments were carried out as indicated. Statistical analysis was performed with one-way or two-way analysis of variance (ANOVA) with Tukey’s multiple comparisons test. Student’s *t*-test was used for TPC and DPPH assays. *P* values less than 0.05 were considered statistically significant. GraphPad Prism 7.0 software (San Diego, CA, USA) was utilised for the analysis.

## 3. Results

### 3.1. Total Phenolic Content (TPC) and 2,2-Diphenyl-1-Picrylhydrazyl (DPPH) Scavenging Activities of Hot Water (HE-HWA) and Ethanolic (HE-ETH) Extracts

**Figure 1:**
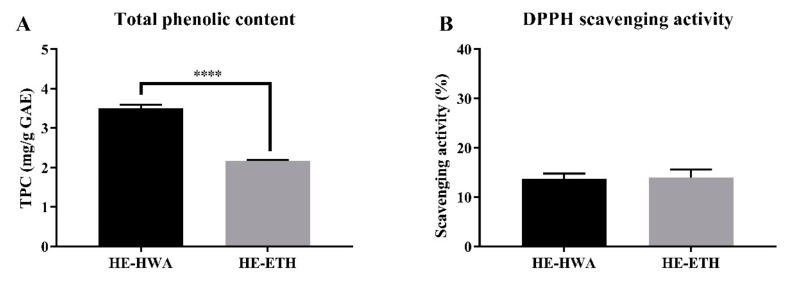
(**A**) Total phenolic content (TPC) and (**B**) 2,2-diphenyl-1-picrylhydrazyl (DPPH) radical scavenging activity of hot water (HE-HWA) and ethanolic (HE-ETH) extracts. Gallic acid equivalent (GAE) was used for relative quantification of the TPCs. In DPPH scavenging assay, the final concentration of HE-HWA and HE-ETH was 1 mg/mL. All values presented correspond to mean ± SD of three independent experiments (*n* = 3). Values of **** *p* < 0.0001 was considered as statistically significant.

Hot water extraction gave a significantly (*p* < 0.0001) higher total phenolic contents (3.50 ± 0.09 mg/g GAE) compared to ethanolic extract with 2.17 ± 0.03 mg/g GAE (Figure 1A). However, their DPPH scavenging abilities were not significantly (*p* > 0.05) different (Figure 1B). HE-HWA at 1 mg/mL scavenged 13.75 ± 1.01% of DPPH free radicals which was comparable to HE-ETH with 13.93 ± 1.64%.

### 3.2. Effects of Hot Water (HE-HWA) and Ethanolic (HE-ETH) Extracts on the Viability of BV2 and HT22

**Figure 2:**
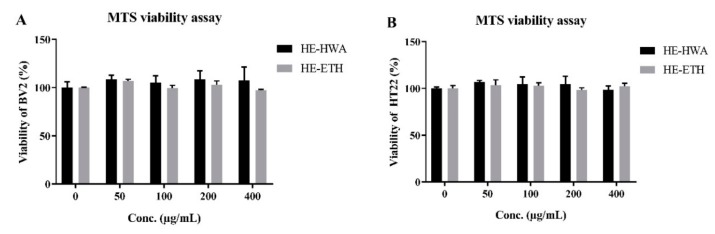
Cytotoxicity of hot water (HE-HWA) and ethanolic (HE-ETH) extracts on (**A**) BV-2 microglia and (**B**) HT22 neurons. The results were expressed as percentage of viable cell versus control, with control considered as 100% cell viability. All values presented correspond to mean ± SD of three independent experiments (*n* = 3).

Prior to the investigation of anti-neuroinflammatory and neuroprotective activities, the effects of HE-HWA and HE-ETH on the viability of BV2 murine microglia and HT22 neurons from mouse hippocampus was assessed by MTS assay to exclude any possibilities of neurotoxicity and proliferative activities. Figure 2A,B shows that HE-HWA and HE-ETH from 50 μg/mL to 400 μg/mL exerted neither inhibition nor increased the viability of BV2 and HT22 (*p* > 0.05). These results suggested that both HE-HWA and HE-ETH were neither neurotoxic nor stimulated the growth of BV2 microglia and HT22 neurons. Both extracts at concentrations ranging from 100–400 μg/mL were then investigated for anti-neuroinflammation and neuroprotection activities.

### 3.3. Effects of Hot Water (HE-HWA) and Ethanolic (HE-ETH) Extracts on Lipopolysaccharide (LPS)-Induced NO Production in BV2

**Figure 3:**
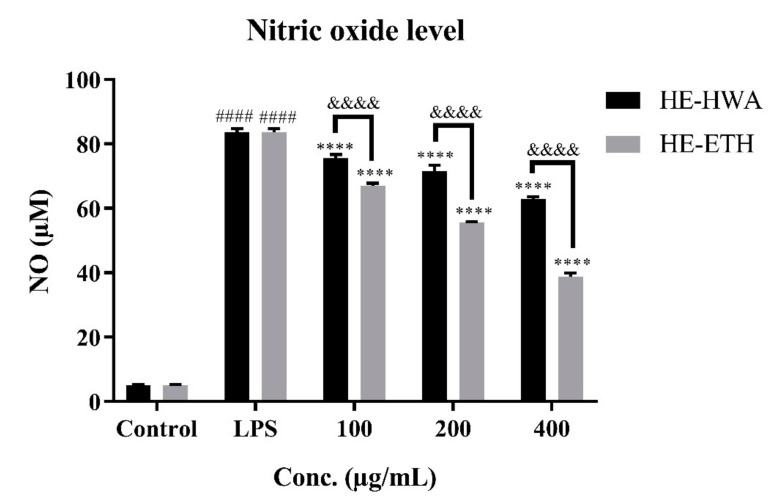
Inhibitory effects of hot water (HE-HWE) and ethanolic (HE-ETH) extracts on LPS-induced NO production in BV-2 microglial cells. All values presented correspond to mean ± SD of three independent experiments (*n* = 3). Values of #### *p* < 0.0001 compared with control and **** *p* < 0.0001 compared with lipopolysaccharide (LPS)-treated group, were considered as statistically significant. Values of &&&& *p* < 0.0001 between the treatment groups were considered as statistically significant.

As shown in Figure 3, LPS significantly (*p* < 0.0001) increased the NO production from 5.06 ± 0.23 μM to 83.68 ± 1.04 μM in BV-2 cells. In contrast, co-incubation with HE-HWA and HE-ETH markedly (*p* < 0.0001) reduced the amount of NO production in dose-dependent manner. Although both HE-HWA and HE-ETH significantly (*p* < 0.0001) suppressed NO accumulation, Sidak’s multiple comparison test revealed that NO reduction ability of HE-ETH was more effective than HE-HWA at 100 μg/mL, 200 μg/mL and 400 μg/mL. The highest NO reduction from 83.68 ± 1.04 μM to 38.73 ± 1.17 μM was accomplished by 400 μg/mL of HE-ETH.

### 3.4. Effects of Hot Water (HE-HWA) and Ethanolic (HE-ETH) Extracts on the Viability of H_2_O_2_-Treated HT22

**Figure 4:**
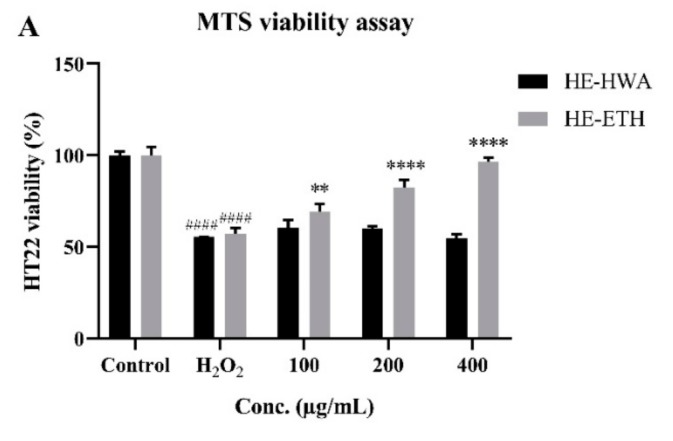

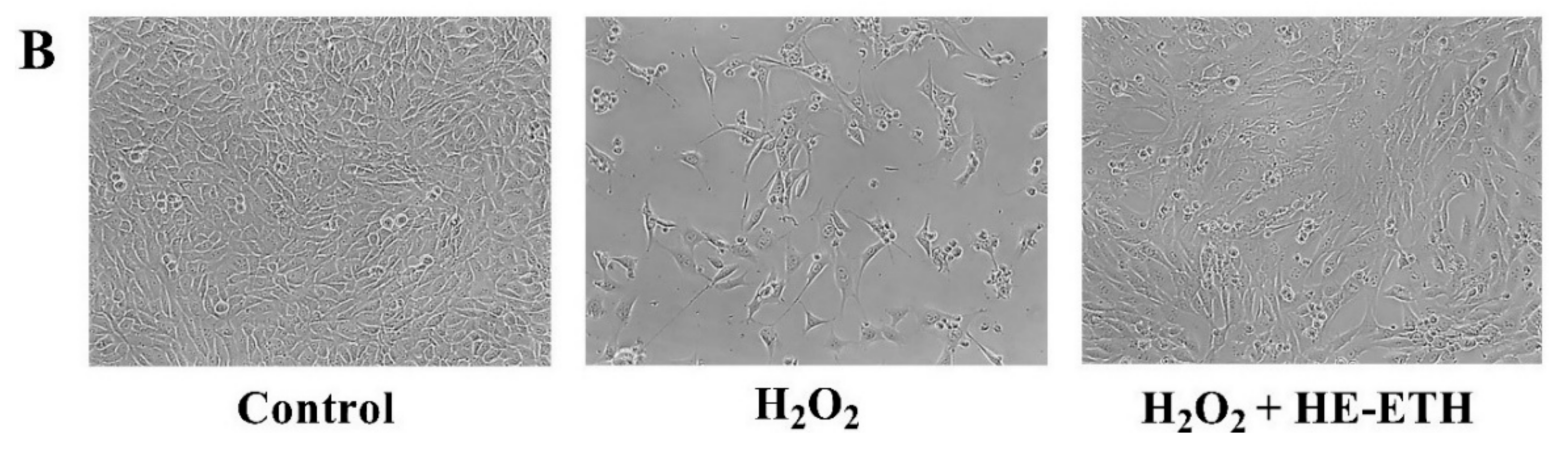
(**A**) Protective effects of hot water (HE-HWA) and ethanolic (HE-ETH) extracts on H_2_O_2_-treated HT22 neurons measured by MTS viability assay and (**B**) morphology of the neurons (control, 250 μM H_2_O_2_ and 250 μM H_2_O_2_ + 400 μg/mL HE-ETH) under light microscope. The results were expressed as percentage of viable cell versus control, with control considered as 100% cell viability. All values presented correspond to mean ± SD of three independent experiments (*n* = 3). Values of #### *p* < 0.0001 compared with control, and ** *p* < 0.01; **** *p* < 0.0001 compared with H_2_O_2_-treated group were considered as statistically significant. Scale bar denotes 100 μm.

The potential of HE-HWA and HE-ETH in protecting HT22 against H_2_O_2_-induced neurotoxicity was investigated by co-incubating the extract at varying concentrations with 250 μM H_2_O_2_. As shown in Figure 4A, the viability of HT22 was significantly (*p* < 0.0001) reduced by 250 μM H_2_O_2_ from 100 ± 4.63% to 57.23 ± 2.95%. However, co-incubation with HE-ETH at 100 μg/mL, 200 μg/mL, and 400 μg/mL provided dose-dependent neuroprotection by significantly (*p* < 0.01, *p* < 0.0001, *p* < 0.0001) improving the viability to 69.04 ± 4.57%, 82.34 ± 4.17%, and 96.75 ± 2.11% respectively. Meanwhile, HE-HWA did not show any neuroprotective activity in HT22.

The morphological observation of HT22 under light microscope further supported the MTS cell viability data (Figure 4B). After 24 h of incubation in 250 μM H_2_O_2_, increased extracellular spaces with notable reduction in number of cells were observed. However, HE-ETH at 400 μg/mL managed to restore the viability of HT22 comparable to the control groups.

### 3.5. Effects of Ethanolic Extract (HE-ETH) on the Antioxidant Activities in H_2_O_2_-Treated HT22

**Figure 5:**
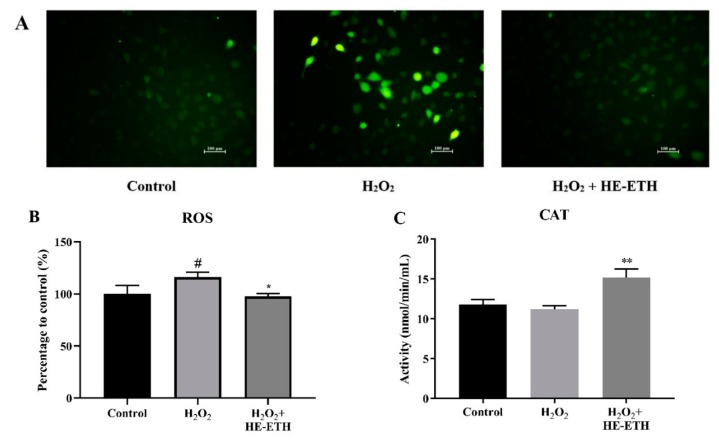

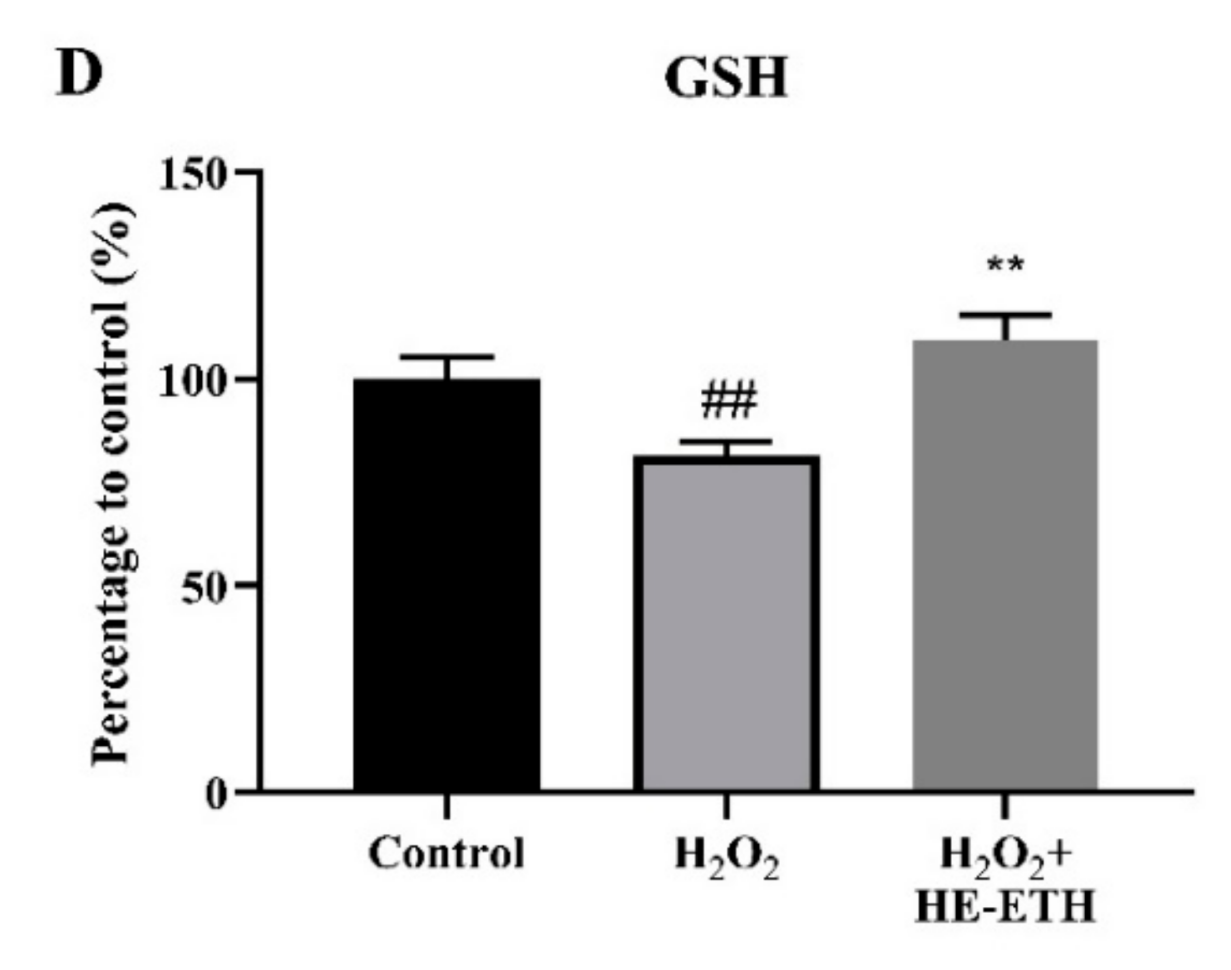
Antioxidant activities of ethanolic extract (HE-ETH) on H_2_O_2_-treated HT22 neurons. HT22 was co-incubated with 250 μM H_2_O_2_ and 400 μg/mL HE-ETH before subjected to series of antioxidant assays. (**A**) Representative images of intracellular reactive oxygen species (ROS) stained by 2’,7’-dichlorodihydrofluorescin diacetate (DCFH-DA) with quantification of (**B**) ROS level, (**C**) catalase (CAT) activity and (**D**) glutathione (GSH) content. All values presented correspond to mean ± SD of triplicates (*n* = 3). Values of # *p* < 0.05; ## *p* < 0.01 compared with control, and * *p* < 0.05; ** *p* < 0.01 compared with H_2_O_2_-treated group were considered as statistically significant. Scale bar denotes 100 μm.

The mechanism of neuroprotection by HE-ETH was further investigated by assessing antioxidant activities in H_2_O_2_-treated HT22 neurons. As 400 μg/mL of HE-ETH earlier demonstrated protective ability, and therefore the concentration was selected for further assays. After 6 h, the application of 250 μM H_2_O_2_ resulted in significantly (*p* < 0.05) higher intensity of green fluorescence of DCF from DCFH-DA that strongly indicates ROS accumulation. Co-incubation with 400 μg/mL HE-ETH markedly (*p* < 0.05) diminished the intensity of green fluorescence from 116.10 ± 4.73% to 97.71 ± 2.66% therefore indicating its ROS-scavenging activity (Figure 5A,B). Although H_2_O_2_ treatment did not lower the CAT activity of HT22, HE-ETH significantly (*p* < 0.01) increased the activity of the antioxidant enzyme from 11.19 ± 0.44 nmol/min/mL to 15.19 ± 1.07 nmol/min/mL (Figure 5C). In addition, H_2_O_2_ treatment significantly (*p* < 0.01) depleted GSH content from 100.00 ± 5.26% to 81.42 ± 3.44% which was significantly increased to 109.40 ± 5.83% by HE-ETH (Figure 5D).

### 3.6. Effects of Ethanolic Extract (HE-ETH) on the Mitochondrial Functions in H_2_O_2_-Treated HT22

**Figure 6:**
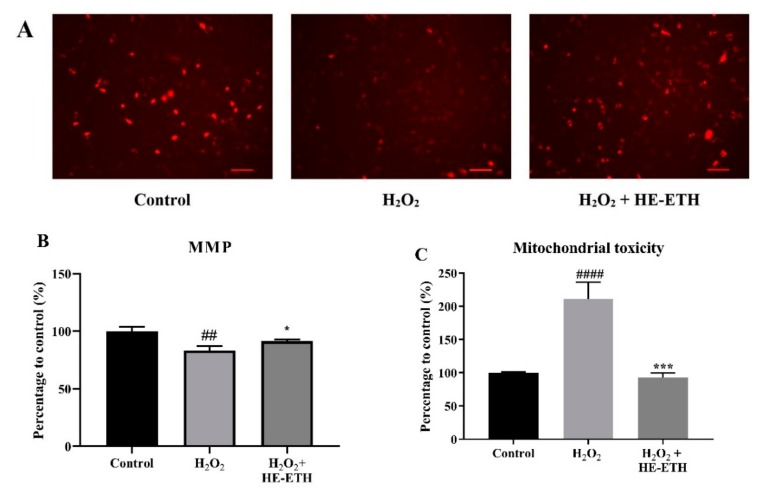

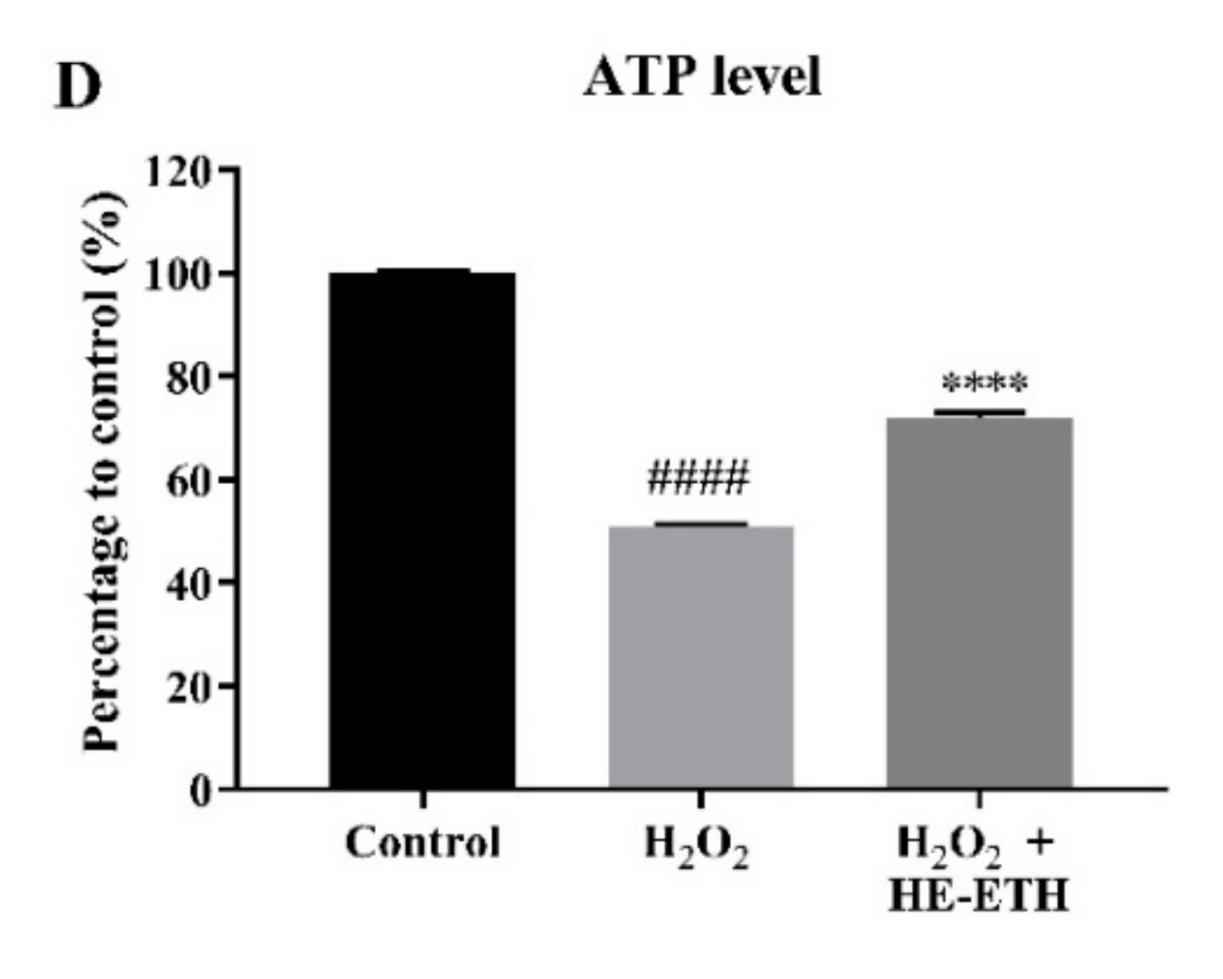
Improvement of mitochondrial functioning by ethanolic extract (HE-ETH) on H_2_O_2_-treated HT22 neurons. HT22 was co-incubated with 250 μM H_2_O_2_ and 400 μg/mL HE-ETH before subjected to series of assays to assess mitochondrial functions. (**A**) Representative images of mitochondrial membrane potential (MMP) stained by tetramethylrhodamine ethyl ester (TMRE) with quantification of (**B**) MMP, (**C**) mitochondrial toxicity and (**D**) ATP level. All values presented correspond to mean ± SD of triplicates (*n* = 3). Values of ## *p* < 0.01; #### *p* < 0.0001 compared with control, and * *p* < 0.05; *** *p* < 0.001; **** *p* < 0.0001 compared with H_2_O_2_-treated group were considered as statistically significant. Scale bar denotes 100 μm.

After 6 h, the application of 250 μM H_2_O_2_ significantly (*p* < 0.05) decreased the intensity of red-orange fluorescence of TMRE from 100.00 ± 3.74% to 83.50 ± 3.70% indicating reduced MMP which was again restored (*p* < 0.05) to 91.42 ± 1.45% by co-incubation with HE-ETH (Figure 6A,B). HE-ETH was also found to significantly (*p* < 0.001) reduce the elevated mitochondrial toxicity from 211.20 ± 25.37% to 92.92 ± 6.95% (Figure 6C). Further, H_2_O_2_ treatment markedly (*p* < 0.0001) decreased ATP content from 100.00 ± 0.22% to 50.74 ± 0.58% which was significantly increased to 72.01 ± 1.05% by HE-ETH (Figure 6D).

### 3.7. Effects of Ethanolic Extract (HE-ETH) on the Anti-Apoptosis in H_2_O_2_-Treated HT22

**Figure 7:**
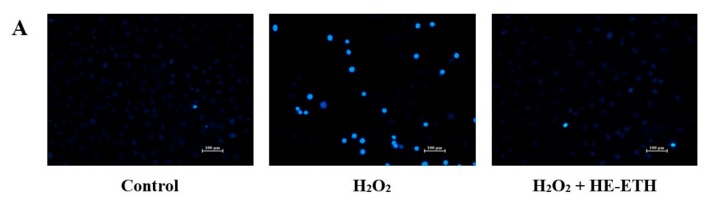

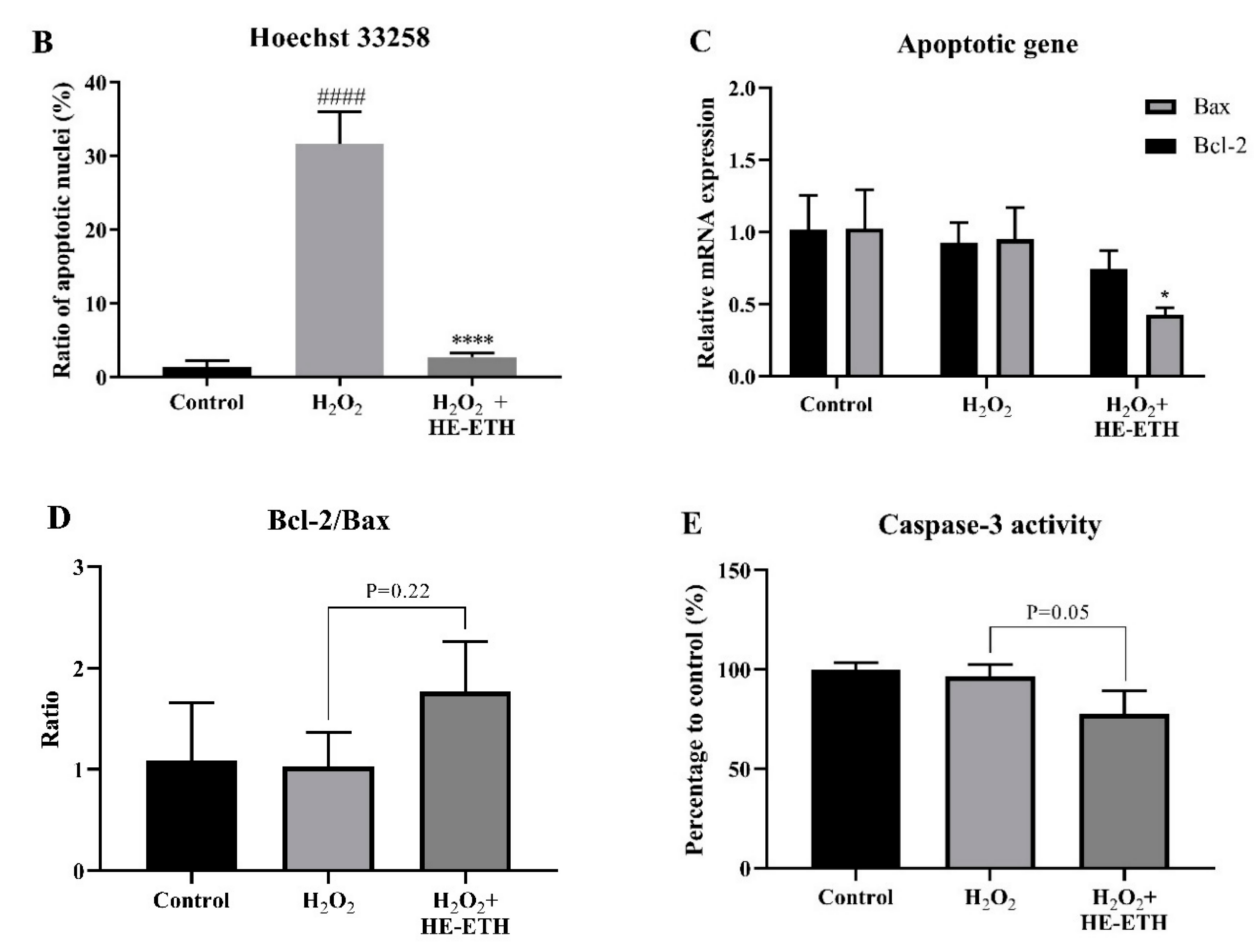
Anti-apoptotic activities of ethanolic extract (HE-ETH) on H_2_O_2_-treated HT22 neurons. HT22 was co-incubated with 250 μM H_2_O_2_ and 400 μg/mL HE-ETH before subjected to apoptosis assays. (**A**) Representative images of apoptotic nuclei stained by Hoechst 33258 with (**B**) ratio of apoptotic nuclei, (**C**) quantification of Bcl-2 and Bax mRNA expression, (**D**) ratio of Bcl-2/Bax and (**E**) caspase 3 activity. All values presented correspond to mean ± SD of triplicates (*n* = 3). Values of #### *p* < 0.0001 compared with control, and * *p* < 0.05; **** *p* < 0.0001 compared with H_2_O_2_-treated group were considered as statistically significant. Scale bar denotes 100 μm.

Apoptotic features were assessed by nuclear labelling with membrane-permeable reagent Hoechst 33,258. The nuclei of control cells were observed as homogeneously dim-blue with regular contours and rounded shapes. After exposure to H_2_O_2_, majority of the nuclei showed bright-blue fluorescence pattern due to chromatin condensation and nuclear fragmentation indicating typical hallmarks for apoptosis. However, co-incubation with HE-ETH inhibited these characteristics of apoptosis (Figure 7A). Quantitative analysis of Hoechst 33,258 revealed that treatment with H_2_O_2_ resulted in 31.63 ± 4.41% apoptotic cells, significantly (*p* < 0.0001) higher compared with 1.43 ± 0.82% of apoptotic cells in control group (Figure 5F). Co-incubation with HE-ETH significantly (*p* < 0.0001) decreased H_2_O_2_-induced apoptotic cells to 2.74 ± 0.58% (Figure 7B).

Based on Figure 7C, H_2_O_2_ did not alter the anti-apoptotic Bcl-2 and pro-apoptotic Bax gene expressions. However, co-incubation with HE-ETH significantly (*p* < 0.05) reduced Bax expression. Ratio of Bcl-2/Bax which is known to indicate the apoptotic state, inversely related to the degree of apoptosis [34] was then calculated. HE-ETH increased the Bcl-2/Bax ratio in H_2_O_2_-treated HT22 cells from 1.03 ± 0.34 to 1.77 ± 0.49 although not statistically significant (*p* > 0.05; *p* = 0.22). In addition, caspase 3 activity which is a hallmark of apoptosis [35,36] was decreased (*p* = 0.05) by HE-ETH (Figure 7E).

### 3.8. Effects of Ethanolic Extract (HE-ETH) on the Transcriptional Expression of Nrf2/NQO1/HO1 Antioxidant Pathway

**Figure 8:**
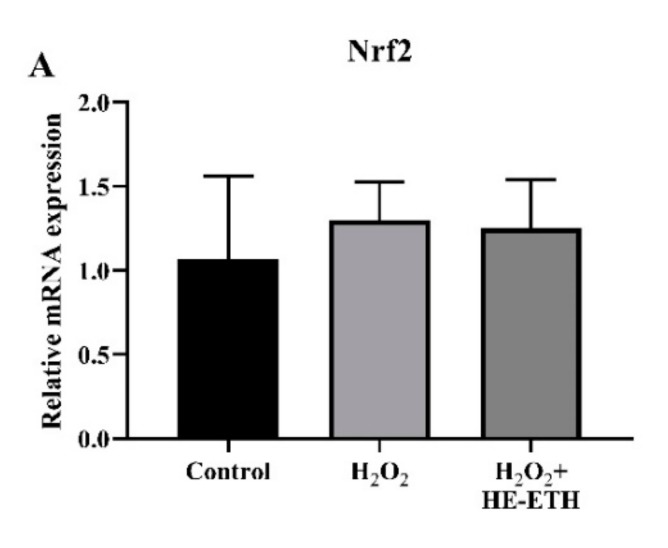

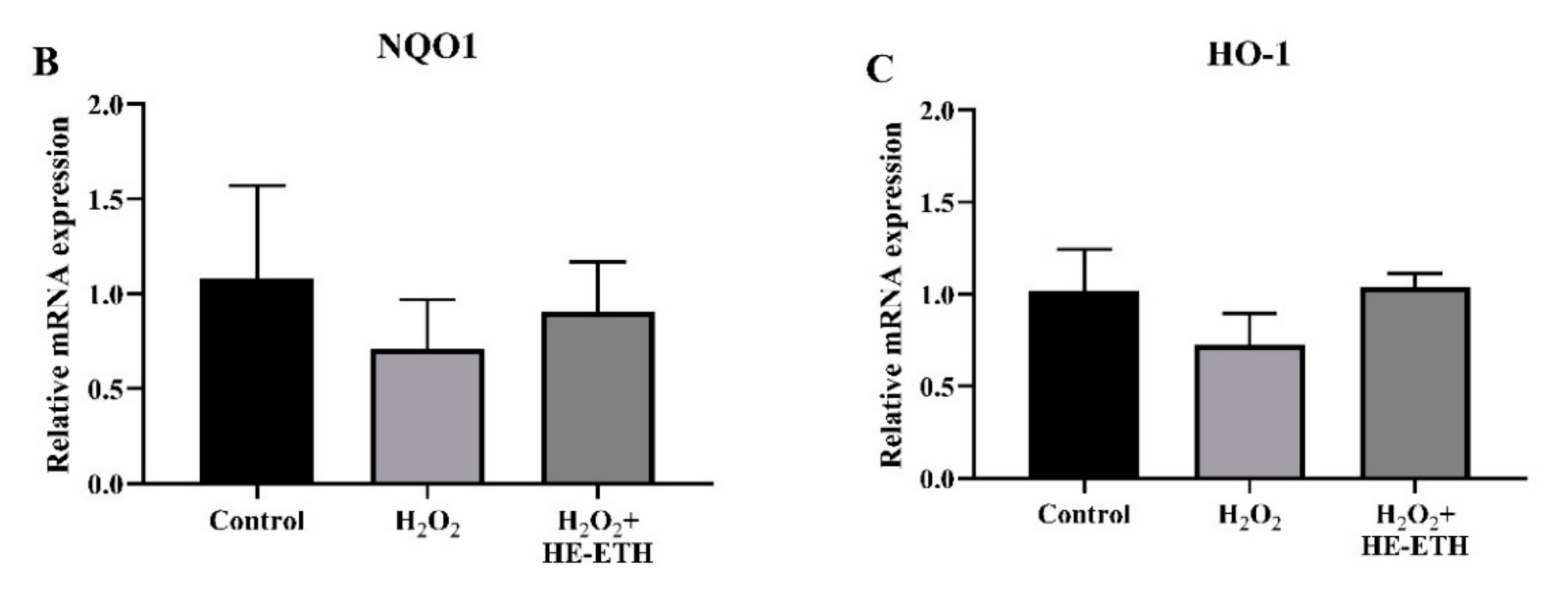
Analysis of transcriptional expression measured by qPCR. HT22 was co-incubated with 250 μM H_2_O_2_ and 400 μg/mL ethanolic extract (HE-ETH) before subjected to qPCR for quantification of (**A**) nuclear factor erythroid 2-related factor 2 (Nrf2), (**B**) NAD(P)H quinone dehydrogenase 1 (NQO1) and (**C**) heme oxygenase-1 (HO-1) mRNA expression. All values presented correspond to mean ± SD of triplicates (*n* = 3).

Analysis by qPCR revealed non-significant (*p* > 0.05) involvement of HE-ETH in regulation of Nrf2 and NQO1 expression (Figure 8A,B). On the other hand, H_2_O_2_ treatment apparently reduced relative HO-1 expression from 1.02 ± 0.23 to 0.73 ± 0.17 although it was not significant (*p* > 0.05; *p* = 0.17). Co-incubation with HE-ETH again resulted in noticeable but not significant (*p* > 0.05; *p* = 0.14) increase in HO-1 expression (Figure 8C).

## 4. Discussion

Mycelium and basidiocarps of *H. erinaceus* are different in term of their nutritional compositions. While *H. erinaceus* is particularly well-known for two classes of neuroactive compounds, namely hericenones and erinacines, the former can be extracted from the basidiocarps while the latter is available in the mycelium [37]. Undoubtedly, cultured mycelium and the isolated erinacines have gained much more interest in neuro-health promotion. Extensive studies have well documented erinacines as potent NGF-stimulating compounds with remarkable neurite outgrowth activities [13,38,39,40,41,42,43]. However, we proved in our previous studies that hot water and ethanolic extracts of the mushroom’s basidiocarps were also beneficial in promoting neurite outgrowth activities in various immortalized neurons and dissociated cells of brain, spinal cord, and retina [44,45]. In addition to this, we managed to show that the ethanolic extract and the isolated hericenones from the basidiocarps enhanced NGF-mediated neurite outgrowth in PC12 cells via MEK/ERK and PI3K-Akt signaling pathways [30]. Hence, we shifted the focus to clarify whether the mushroom basidiocarps could also be beneficial for neuroprotection. As basidiocarps are what is available to people in the market, they can easily become a preference in one’s diet. In order for society to reap the benefits of consuming the mushroom, it has to be available at its therapeutic potential and the preparation process is within their capability.

In terms of the extraction method, hot water extraction was preferred to yield polysaccharides and other heat stable components [46,47]. Meanwhile, ethanol extraction is generally more favored as it can be used to extract secondary metabolites especially terpenoids including hericenones in *H. erinaceus* [22]. Despite its significantly higher TPC, the DPPH scavenging activity of HE-HWA in this study was similar to that of HE-ETH. The main issue with screening of TPC and DPPH scavenging activity is always the solubility. It is well-known that TPC is a water-based assay, while DPPH assay is based in ethanol. Hence, HE-HWA which is water soluble could give a higher TPC but lower DPPH scavenging activity due to their solvent preferences [48]. In addition, hot water extraction as the name suggests, could deactivate or degrade the heat-labile antioxidant components hence explain why HE-HWA did not get the better of HE-ETH in DPPH scavenging assay regardless of its higher TPC [49,50,51].

Microglia-mediated neuroinflammation is considered a major source of reactive oxygen species (ROS) and reactive nitrogen species (RNS) in the brain and plays a significant role in the pathogenesis of neurodegenerative diseases [52]. Microglial cells constitute the brain’s defence system by inducing phagocytosis and regulating the release of pro-inflammatory cytokines [45,46]. However, chronic and excessive induction of pro-inflammatory mechanisms by activated microglia will lead to overproduction of potentially neurotoxic cytokines, ROS and RNS; including NO and H_2_O_2_ [53,54,55,56]. Despite its’ role as both neuromodulator and neurotransmitter in the central nervous system (CNS), the overproduction of NO has been implicated in neuroinflammation and subsequent neuronal death in neurodegenerative diseases [57]. In our study, HE-HWA and HE-ETH from the mushroom’s basidiocarps were shown to reduce the NO level in LPS-activated BV2 microglia. However, the percentage reduction of NO was more immense by HE-ETH at every concentration tested. A huge number of studies reported anti-inflammatory activities of *H. erinaceus* by suppressing the release of NO in RAW 264.7 murine macrophage from blood [24,25,26,27,28]. To the best of our knowledge, this was the first report showing NO-suppressing activities by *H. erinaceus* in BV2 microglia indicating its potential in ameliorating chronic inflammation in the CNS. Nevertheless, the anti-inflammatory activities need to be further clarified by investigating the release of other important pro-inflammatory cytokines such as tumor necrosis factor alpha (TNFα) and interleukins as well as inflammatory mediators including inducible nitric oxide synthase (iNOS), cyclooxygenase (COX), and prostaglandin E2 (PGE2).

Hydrogen peroxide (H_2_O_2_), a highly reactive ROS, is one of the most important mediators of oxidative stress proven to induce neuronal dysfunction through the activation of mitochondria related apoptotic signals [58,59]. H_2_O_2_ has been widely utilised as a neurotoxic challenge paradigm to mimic in vitro oxidative stress in many different cell types including murine hippocampal HT22 cells [60,61,62]. In this study, we demonstrated the ability of HE-ETH to successfully protect HT22 neurons from H_2_O_2_-induced neurotoxicity. Our study further elucidated the mechanisms of neuroprotection by HE-ETH in HT22 through improvement of antioxidant, mitochondrial functioning, and anti-apoptosis. Although *H. erinaceus* was previously demonstrated to stimulate neuroprotection in various model of induced neurotoxicity, this was the first report showing its ability in H_2_O_2_-induced oxidative neurotoxicity. Other reports utilized different neurotoxic models including glutamate, amyloid beta, and MPTP to assess neuroprotective effects of the mushrooms [19,20,21,22,23]. An attempt to show neuroprotective activity of *H. erinaceus* against H_2_O_2_ in NG108-15 neuroblastoma-glioma cell line was non successful [29]. Instead, a study conducted in human gastric mucosa epithelium cell revealed the ability of an isolated polysaccharide, EP-1 from mycelium of *H. erinaceus* to prevent H_2_O_2_-induced toxicity death by inhibiting activation of apoptotic cellular signals within mitochondria-dependent apoptosis [46]. This was in accordance with our study, which demonstrated the role of similar mechanisms in the promotion of neuronal viability.

Diverse studies have reported that mushrooms possess bioactive compounds that enhance various antioxidant enzymes in the body including CAT, GSH, and superoxide dismutase (SOD). These enzymes act by catalyzing reactions that neutralize free radicals and ROS in order to protect cells from damage [63,64]. CAT is a primary defence unit against oxidative toxicity, responsible for breakdown of H_2_O_2_ to O_2_ (47). Numerous mushrooms such as *Ganoderma lucidum*, *Pleurotus cystidiosus*, *P. ostreatus*, *P. djamor*, and *P. eryngii* were effective in elevating CAT activity, hence preventing H_2_O_2_-induced toxicity [63,65,66,67,68,69]. In addition, several mushrooms including *Agaricus bisporus, G. lucidum* and *P. ostreatus* demonstrated the GSH-related activities. GSH, often referred to as a master antioxidant compound is crucial to block reactive hydroxyl free radicals and oxygen-centred free radicals hence protecting cells from toxicity [65,68,69,70,71]. Hence, the significant reduction of ROS in this study could be attributed to the elevated level of CAT activity and GSH content.

Antioxidant, mitochondrial health and anti-apoptosis are always interrelated in overcoming oxidative stress-induced toxicity, hence becoming the main targets of neuroprotective therapy [72,73,74,75,76]. Similarly, we found in this study that the increase in antioxidant activities were accompanied by improvement of MMP and reduction of mitochondrial toxicity. This was reflected by the increase in ATP content, an indicator of mitochondrial health, and subsequent nuclear apoptosis and reduction in cellular viability. Just recently, the polysaccharide from *H. erinaceus* mycelium was found to protect ulcerative colitis-induced rats and Caco-2 cells by counteracting oxidative stress and improving mitochondrial function [47]. A study using the hot water extract reported its significant neuroprotective properties in glutamate-induced toxicity in PC12 cells. The extract was found to prevent nuclear apoptosis by suppression of intracellular ROS accumulation and MMP depolarization [23]. On the other hand, erinacine A-enriched ethanolic extract from *H. erinaceus* mycelium was shown to exert prominent anti-apoptotic activity via ROS-caspase dependent pathway against glutamate-insult in PC-12 cells [22]. Nevertheless, all those studies above mentioned mycelium as the source for extraction rather than basidiocarps of the mushroom, which were used in our study.

Under normal circumstances, Nrf2 is localized in the cytoplasm and bond with its inhibitor, Kelch-like ECH associated protein 1 (Keap1). Once stimulated with oxidative stress, Nrf2 translocate into the nucleus to binds with the anti-oxidant response elements (AREs) and regulate the expression of antioxidant genes, primarily HO-1 and NQO1 [77,78]. HO-1 is an inducible enzyme that catalyzes the degradation of heme into carbon monoxide (CO), biliverdin, and free iron, which exert anti-oxidative functions via various mechanisms including reduction of ROS production [79,80,81]. Meanwhile, NQO1 functions as quinone reductase by reducing quinones to hydroquinones, hence preventing the one electron reduction of quinones that results in the production of radical species [82,83]. The Nrf2-ARE signaling pathway is actively involved in various CNS diseases including ischemic stroke [84], traumatic brain injury [85], Parkinson’s disease (PD) [86], and Alzheimer’s disease [87]. In this study, we witnessed non-significant enhancement of Nrf2, NQO1, and HO-1 transcriptional expression by HE-ETH. However, it cannot be concluded that Nrf2-ARE pathway was not involved, because CAT and GSH, which are also by-products of Nrf2/ARE activation [88], were significantly enhanced by HE-ETH. Therefore, further studies need to be conducted to comprehensively elucidate the involvement of Nrf2/ARE pathway by suitable approaches, such as overexpression of Keap1 or transient transfection of Nrf2–small interfering RNA (siRNA) [89]. Since HE-ETH and its constituents were found to induce NGF synthesis and upregulation of MAPK and Akt pathways [30] which were proven elsewhere to be neuroprotective [90,91,92,93], it is worthy to explore whether those pathways are also responsible for the activities of HE-ETH in this study.

## 5. Conclusions

HE-ETH compared to HE-HWA exerted potent neuroprotection and higher NO-suppressing anti-inflammatory activity in HT22 hippocampal neurons and BV2 microglia in vitro. The mechanisms of neuroprotection were through the improvement of antioxidant, mitochondrial function and anti-apoptosis. HE-ETH could be potentially explored as a neuroprotective agent in neuron-glia environment. However, further studies are needed to elucidate the mechanistic pathways involved in the neuroprotection and to study its efficacy in vivo.
